# Cytomegalovirus-Induced Optic Neuritis Through Cerebrospinal Fluid Viral Transmission in an Immunocompetent Patient: A Case Report

**DOI:** 10.1097/WNO.0000000000001834

**Published:** 2023-04-19

**Authors:** Shizuka Takahashi, Noriyasu Hashida, Kazuichi Maruyama, Rina Omura, Rei Sakurai, Takeshi Morimoto, Kohji Nishida

**Affiliations:** Department of Ophthalmology (ST, NH, KM, RO, TM, KN), Osaka University Graduate School of Medicine, Osaka, Japan; Department of Ophthalmology (ST), Higashiosaka City Medical Center, Osaka, Japan; Integrated Frontier Research for Medical Science Division (KM, KN), Institute for Open and Transdisciplinary Research Initiatives (OTRI), Osaka University, Osaka, Japan; Advanced Visual Neuroscience (TM), Osaka University Graduate School of Medicine, Osaka, Japan; and Department of Neurology (RS), Osaka University Graduate School of Medicine, Osaka, Japan.

Clinical findings, such as corneal endothelitis, iridocyclitis, retinitis,^1^ and papillitis,^2^ are observed in the cytomegalovirus (CMV)-infected eyes not only in immunocompromised hosts but also in immunocompetent hosts. Infection is often binocular in immunocompetent hosts, but the pathway of propagation from one eye to the other is unclear. We report the case of optic neuritis in the left eye (OS) induced by virus-infected cerebrospinal fluid (CSF) transmission from the CMV retinitis–bearing right eye (OD) in an immunocompetent patient.

**FIG. 1.**
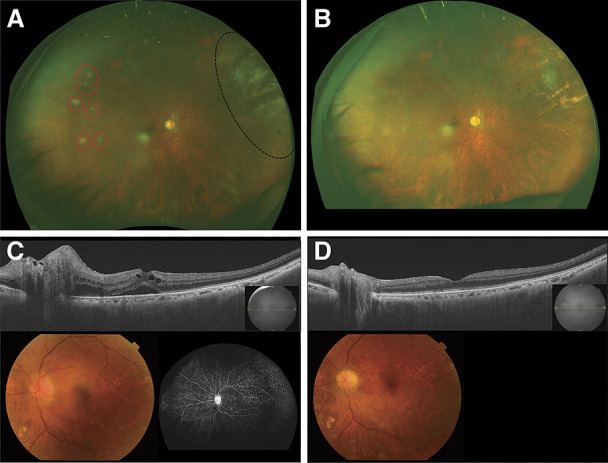
Fundus images of a patient with retinitis and optic neuritis at onset and after treatment. **A**. Fundus photograph of the retinitis at onset. Fundus photograph of the retinitis eye was obtained at onset. The red dotted circles indicate white chorioretinal spots, and the black dotted circle indicates postoperative scarring from the retinal detachment. **B**. Fundus photograph of the retinitis after treatment. The white chorioretinal spots have become atrophic after antiviral therapy. **C**. Fundus images of the optic neuritis at onset. The upper row shows optical coherence tomography (OCT) image at the onset of optic neuritis, which shows the swollen optic nerve and subfoveal serous detachment at the macula. The lower left image is a fundus photograph and the lower right is early-phase fluorescein angiographic (FA) image, indicating the hyperfluorescenced optic nerve head. **D**. Fundus images of the optic neuritis after treatment. The upper row is an OCT image obtained after antiviral and high-dose corticosteroid therapy. The optic nerve swelling has almost disappeared, and the macula is almost normal. The lower right is a fundus photograph, showing that the optic disc head from 2 o'clock to 4 o'clock has become pale. FA is not available.

A 63-year-old man complained of blurred vision in the OD. He had a history of cataract surgery of the OD a year ago, intrascleral fixation of an intraocular lens 8 months ago, and a second vitrectomy for retinal detachment 2 months ago. His best-corrected visual acuity (BCVA) was 20/63 OD and 20/25 OS. His intraocular pressure (IOP) was 23 mm Hg OD and 14 mm Hg OS. On slit-lamp examination, the OD had pigmented, spiny keratic precipitates and 2+ anterior chamber cells. Fundoscopy revealed white chorioretinal spots scattered from the equator of the retina to the periphery and postoperative scarring due to the retinal detachment in the superior nasal retina (Fig. 1A). A total of 6.06 × 10^4^ copies/mL CMV in the aqueous humor was detected by polymerase chain reaction (PCR) analysis, whereas no other infectious agents were detected. The laboratory examination showed slightly increased white blood cells (9.57 × 10^3^/μL), negative C-reactive protein, 6.7% HbA1c, neutrophils 55%, lymphocytes 36.4%, monocytes 5.2%, eosinophils 2.8%, basophils 0.6%, negative CMV antigen, and no other abnormalities. The CD4 T lymphocyte was not available. Toxoplasma IgM and IgG, syphilis, human T-lymphotropic virus 1, and HIV serology results were negative. Computed tomographic scans from the chest to the pelvis and brain MRI including orbital views showed no significant findings. He underwent evaluation with an immunology specialist, and no immunodeficiency was identified. Based on the positive PCR testing, CMV retinitis was diagnosed and we administered oral valganciclovir, 900 mg twice daily. However, it was decreased to 450 mg due to severe diarrhea, nevertheless leading to successful remission. The atrophied white spots were observed in the fundus photograph after treatment (Fig. 1B); however, visual acuity in the OD did not improve.

**FIG. 2.**
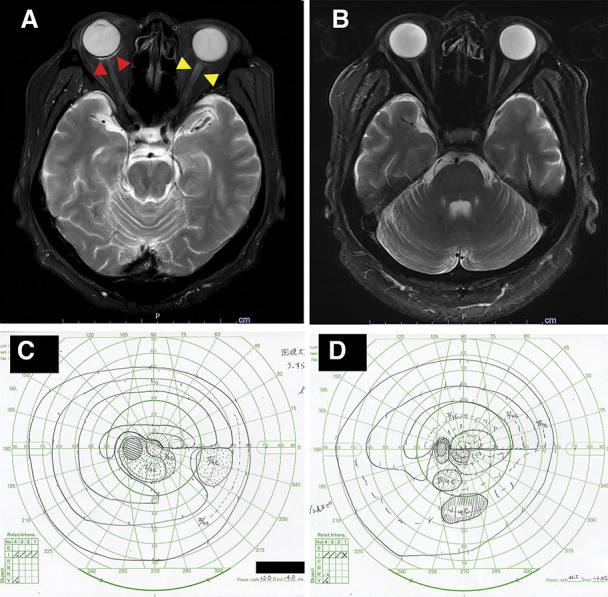
Magnetic resonance imaging and kinetic perimetry of optic neuritis at onset and after treatment. **A**. MRI at onset. Fat-saturated T2-weighted MRI shows hyperintensity along the sclera in the right retrobulbar (*red arrowheads*), suggesting inflammation, and a longitudinal enhancing lesion of the left orbital nerve (*yellow arrowheads*), suggesting optic neuritis. No findings are suggestive of meningitis. **B**. MRI after treatment. The T2-weighted fat-saturated image shows the subtle residual high signal along the sclera in the right retrobulbar and the normal signal near the papilla of the left optic nerve. **C**. GP at onset. Kinetic perimetry shows the lower horizontal hemianopia. **D**. GP after treatment. The lower horizontal hemianopia has narrowed.

Subsequently, he complained of left blurred vision 7 weeks after the remission of the CMV retinitis-bearing OD. His BCVA worsened to 20/125 OD and 20/320 OS. The IOP was 19 mm Hg OD and 14 mm Hg OS. Slit-lamp examination revealed no inflammation in the anterior chamber of either eye. Fundoscopy revealed optic disc swelling in the OS. Optical coherence tomography showed even subfoveal subretinal fluids (SRF) (Fig. 1C). Fat-saturated T2-weighted MRI showed a longitudinal enhancing lesion of the left orbital nerve, suggesting optic neuritis (Fig. 2A). The critical fusion frequency (CFF) was 30-30 Hz OD but only 7-7 Hz OS. Kinetic perimetry showed lower horizontal hemianopia in the OS (Fig. 2C). PCR of the aqueous humor for CMV revealed 1.30 × 10^4^ copies/mL in the OD and was negative in the OS. PCR of CSF was positive for CMV. Blood CMV antigen was negative. Therefore, he was diagnosed with viral optic neuritis in the OS and treated with intravenous ganciclovir for 3 weeks and 2 courses of 3-day high-dose corticosteroids (1 g methylprednisolone), leading to negative CSF CMV by PCR and rapid resolving of SRF (Fig. 1D). His BCVA improved to 20/40 OD and 20/63 OS, and the visual field impairment partially recovered, accompanied by the improved MRI (Fig. 2B, D). The reduced CFF OS returned to 31–32.7 Hz which was within the normal limit.

There are several reports of CMV-induced papillitis in immunocompetent patients,^2,3^ but to the best of our knowledge, there has been no reported case of retinal-localized CMV tracing along the optic nerve and developing optic neuritis in an immunocompetent patient. We report a case of a patient who initially developed CMV retinitis and subsequently developed optic neuritis in the opposite eye due to viral transmission through the CSF. In acute retinal necrosis (ARN), a form of bilateral retinitis caused by the herpes virus, transferring of the virus from one eye to the other may occur by the optic nerve and central nervous system, consistent with the delayed occurrence in the contralateral eye over time.^4^ Generally, CMV uses both monocytic cells and polymorphonuclear cells to disseminate throughout the body.^5^ In this case, however, the transmission pathway of CMV may be similar to the pathway of the herpes virus in ARN. We speculate that the inflammation associated with CMV retinitis spread to the optic nerve, allowing CMV to infiltrate the CSF and transfer to the optic nerve on the opposite side, causing optic neuritis. The negative systemic blood CMV antigen status and the nonoccurrence of synchronous binocular optic neuritis support this infection pathway. Therefore, CMV-infected leukocytes in the circulating blood were not detected, and the blood–cerebrospinal fluid barrier was also maintained.

In this case, the difference in response to treatment between OD and OS is also noteworthy. Retinitis in the OD was rapidly resolved by the administration of antiviral drugs, but optic neuritis in the OS required an extended regimen of antiviral drugs and high-dose steroids. We speculate that this difference is due to a difference in whether the local blood–retinal barrier (BRB) has collapsed. Because of the local onset of CMV retinitis in the OD, the BRB at the retina OD had collapsed. The rapid remission of retinitis also supports the speculation that the antiviral drugs rapidly infiltrated through the broken BRB. On the other hand, the BRB and blood–brain barrier (BBB) around the left optic nerve might have remained intact, such that retinitis did not develop, and inflammation of the optic nerve was prolonged and required high-dose steroids. Thus, steroids are generally effective in idiopathic optic neuritis in which the BBB is broken, but viral optic neuritis with the incomplete collapse of the BBB might be resistant to drugs.

In conclusion, when binocular CMV neuroretinal manifestations develop asynchronously in an immunocompetent patient, CSF PCR may be helpful to confirm the presence of CMV. Prompt diagnosis is crucial for early and proper treatment, recognizing CMV illustrates the propensity to track along axons.
